# 1-Nitropyrene induces acute lung injury via SYVN1/Caspase-11-mediated apoptosis and pyroptosis in pulmonary epithelial cells

**DOI:** 10.3389/fphar.2026.1723593

**Published:** 2026-02-09

**Authors:** Zhao-Lei Ding, Yi-Chen Ma, Chuan-Mei Liu, Xiu Lu, Rong-Rong Wang, Min-Min Tang, Dong-Xu Hua, Ling Zheng, Hui Zhao, Lin Fu

**Affiliations:** 1 Department of Respiratory and Critical Care Medicine, The Second Affiliated Hospital of Anhui Medical University, Hefei, Anhui, China; 2 Institute of Respiratory Diseases, The Second Affiliated Hospital of Anhui Medical University, Hefei, Anhui, China; 3 Department of Respiration, The First Affiliated Hospital of Shandong Second Medical University (Weifang People’ Hospital), Weifang, Shandong, China; 4 Center for Big Data and Population Health of IHM, The Second Affiliated Hospital of Anhui Medical University, Hefei, Anhui, China

**Keywords:** 1-Nitropyrene, acute lung injury, apoptosis, caspase-11, pyroptosis, SYVN1

## Abstract

**Background:**

1-Nitropyrene (1-NP) is harmful to the respiratory system and can evoke acute lung injury (ALI). Pyroptosis and apoptosis, two important types of programmed cell death, are involved in the pathological process of ALI. However, the roles and mechanisms of pyroptosis and apoptosis on 1-NP-incurred ALI remain unclear.

**Methods:**

All the mice were exposed to a single dose of 1-NP (20 μg/mouse, dissolved in saline) or normal saline via intratracheal instillation. At different times after 1-NP exposure, the mice were sacrificed. Mouse lung epithelial (MLE-12) cells were incubated with 1-NP (5 μM), the indicators of pyroptosis and apoptosis were detected.

**Results:**

Pulmonary pathological injury and inflammatory cell infiltration was observed in 1-NP-exposed mice. Additionally, the indicators of apoptosis, Bcl-2 was downregulated, Bad and Caspase-3, and apoptotic cells were increased in 1-NP-exposed mouse lungs and mouse lung epithelial (MLE-12) cells. Meanwhile, the proteins of GSDMD and Pro- and Cleaved Caspase-11 and the mRNAs of *Il-1β* and *Il-18*, which are markers of pyroptosis, were increased after 1-NP treatment. Moreover, pretreatment with wedelolactone (WED), an antagonist of Caspase-11, alleviated 1-NP-induced ALI. As expected, pharmacological inhibition or genetic deletion of Caspase-11 abolished 1-NP-induced apoptosis and pyroptosis. Interestingly, 1-NP attenuated Caspase-11 proteasome degradation. Mechanistically, 1-NP downregulated the expression of SYVN1, an E3 ubiquitin ligase of Caspase-11. 1-NP promoted the interaction between SYVN1 and Caspase-11 and inhibited Caspase-11 ubiquitination and subsequent proteasome degradation. Transfection with SYVN1 overexpression plasmids abolished 1-NP-mediated the reduction of Caspase-11 ubiquitination-dependent degradation, apoptosis, and pyroptosis.

**Conclusions:**

These results revealed that acute 1-NP may induce ALI via Caspase-11-mediated apoptosis and pyroptosis by downregulating SYVN1.

## 1 Introduction

With rapid socioeconomic progress and the increase in industrialization, environmental pollution, which adversely affects the entire world, has attracted increasing attention. A 2016 report indicates more than 90% of urban residents live in indoor environments with poor air quality, and these conditions contribute to two out of three deaths worldwide (World Health Organization, 2016). 1-Nitropyrene (1-NP), a representative pollutant of nitro-polycyclic aromatic hydrocarbons (nitro-PAHs), is prevalent atmospheric pollutants and derived primarily from the incomplete combustion of carbonaceous organic substances, fine particulate matter, and other compounds, and it is a danger to public health (Schauer et al., 2004; Gao et al., 2018). Furthermore, 1-NP has already been found in river water and food (Deng and Chan, 2017; Ohe and Nukaya, 1996).

Exposure to 1-NP is associated with many diseases, including testicular steroidogenesis dysfunction (Li et al., 2023), DNA damage in the heart (Zhao et al., 2019), anxiety-like behaviour (Wang et al., 2023), and impaired embryo implantation (Liang et al., 2021). Previous studies from our laboratory demonstrated that chronic 1-NP initiates pulmonary fibrosis and chronic obstructive pulmonary disease (COPD) (Fu et al., 2021; Li S. R. et al., 2024; Wang et al., 2025). Despite its harmfulness, the influence of acute 1-NP on the respiratory system remains inadequately characterized. Acute lung injury (ALI), with very high incidence rates worldwide, is a serious respiratory disease accompanied by severe inflammatory reactions, and it can progress to acute respiratory dysfunction syndrome (ARDS), which is an emergency situation (Mowery et al., 2020; Fan et al., 2018). ALI has multiple aetiologies, such as endogenous and exogenous pathogenic factors, and may evoke respiratory failure and death (Beitler et al., 2022; Butt et al., 2016). There are more than 3 million cases of ALI and 75,000 related deaths reported annually (Yadav et al., 2017). Despite advances in detection methods and treatments, the mortality rate is still 40–60% in intensive care units (Dres et al., 2018). The previous studies have uncovered that air pollution elevates the susceptibility to ARDS (Bennett and Reilly, 2024; Reilly et al., 2019). In addition, many animal experiments suggested that exposure to environmental pollutions is one of the important risks of ALI (Su et al., 2025; Gao et al., 2025). Although the previous study from our laboratory has unveiled that acute 1-NP exposure induces pulmonary inflammation and ALI in mice (Hu et al., 2020), little is known about the specific mechanism of 1-NP-induced ALI. Hence, there is an urgent need to elucidate the pathogenesis of ALI and explore available therapeutic drugs.

Programmed cell death is a central process of innate immunity to protect the host from the infection of pathogenic microorganisms. Pyroptosis and apoptosis, two important types of programmed cell death, have different physiological and pathological functions and can regulate the cellular lifespan (Bertheloot D, et al., 2021). It’s known that cysteine aspartate-specific protease (Caspase)-11 mediates the non-canonical inflammasome that is activated by various Gram-negative bacterial infections and initiate inflammation signalling and facilitate inflammatory cell death (pyroptosis) (Kayagaki et al., 2011; Broz et al., 2012). Activated Caspase-11 can cleave Gasdermin D (GSDMD), the vital driver of pyroptosis, and separate the N-terminal pore-forming domain which triggers pyroptosis (Burdette et al., 2021). In addition, the evidence has hinted that Caspase-11 can evoke apoptosis via activating Caspase-3 (Kang et al., 2002). An increasing number of investigations have reported that pyroptosis and apoptosis are involved in the pathological process of ALI (Liu et al., 2022; Zhu et al., 2023). In addition, 1-NP exposure incurs apoptosis in human bronchial cells (Kim et al., 2025). However, the exact roles of pyroptosis and apoptosis on acute 1-NP-induced ALI and the physiologic mechanisms remained largely unknown. In consideration of the dual roles of Caspase-11 on pyroptosis and apoptosis, we speculated that Caspase-11 may involve in the progression of 1-NP-mediated pyroptosis and apoptosis. The aim was to explore the roles of pyroptosis and apoptosis on acute 1-NP-induced ALI and the potential mechanisms through animal and cellular experiments. Our research provided new insight into and evidence for the mechanisms underlying environmental pollutant-induced ALI and possible targets for the treatment and prevention of ALI.

## 2 Methods

### 2.1 Chemicals and reagents

1-NP (Cat#N22959) was purchased from Sigma-Aldrich (St. Louis, United States). Cycloheximide (CHX, Cat#C112766) was purchased from Aladdin (Shanghai, China). MG132 (Cat#HY-13259), bafilomycin A1 (BfnA1, Cat#HY-13259), and Wedelolactone (WED, Cat#HY-N0551) were purchased in Med Chem Express (New Jersey, United States). Enhanced chemiluminescence kit and TRIzol reagent were from Thermo Fisher Scientific (MA, United States). The primary antibodies used in this study were listed in Supplementary Table S1.

### 2.2 Animal study design and ALI model establishment

Seven to eight-week-old male mice (C57BL/6J, 22–25 g) were maintained in the animal room with a standard diet and allowed to eat and drink freely. Animal experiments were designed as follow: In Experiment 1, to establish the model of ALI, 40 mice were randomly divided into the control (Ctrl) and 1-NP groups. All the mice were intratracheally instilled with a single dose of 1-NP (20 μg/mouse, dissolved in saline) or normal saline (Hu et al., 2020). At 12, 24, and 48 h (h) after 1-NP intratracheal instillation, the mice were euthanized. Serum samples and lung tissues were collected. In Experiment 2, to assess the role of Caspase-11 on acute 1-NP-evoked ALI in mice, WED, an antagonist of Caspase-11, was used. WED (20 mg/kg/day) was administered by oral gavage at 24 h before acute 1-NP exposure. The Ctrl and 1-NP groups were treated with saline. Then, 1-NP exposure was implemented. At different times after 1-NP exposure, lung samples were taken.

### 2.3 Cell experiments

Mouse lung epithelial (MLE-12) cells were purchased from American Type Culture Collection (ATCC). MLE-12 cells were cultured in high-glucose Dulbecco’s Modified Eagle Medium (DMEM). When the cell density reached 60%, the cells were exposed to 1-NP (5 μM) (Li S. R. et al., 2024). After 1-NP treatment, pyroptosis and apoptosis were detected in MLE-12 cells.

### 2.4 Histology, immunohistochemistry (IHC), and immunofluorescence (IF)

Lung tissues were collected from the mice, immediately fixed and embedded. Lung structure and pathological injury were assessed via haematoxylin and eosin (H&E) staining. The pathological score was estimated on the basis of previous research (Fei et al., 2019). For immunohistochemistry (IHC), pulmonary sections were dewaxed, hydrated, and quenched. Then, antigen repair was executed. Fetal bovine serum (FBS) was used as a blocking agent, and the tissues were incubated with corresponding antibodies. Then, the nucleus was stained with haematoxylin. For immunofluorescence (IF), MLE-12 cells grown on cell climbing sheets or frozen tissue sections were fixed with formalin. Then, surfactant proteins C (SP-C), Caspase-3, or Gasdermin D (GSDMD) primary antibodies, and fluorescent secondary antibodies were added successively. Finally, the nucleus was stained with Hoechst. Positive staining was observed, and images were taken through a microscope.

### 2.5 Western blot assay

Fresh lung tissues or MLE-12 cells were broken using a sonicator. The homogenate was centrifuged, and the concentration of the supernatant was measured (Wang et al., 2025). After electrophoresis, the proteins were transferred to a polyvinylidene fluoride (PVDF) membrane. The membrane was blocked and subsequently incubated with primary and secondary antibodies. Finally, the band signals were determined with an enhanced chemiluminescence kit. Finally, analyses of grey intensity were performed via Odyssey DLx (9142, United States).

### 2.6 Plasmids and transfection

The full-length synoviolin (SYVN1) sequence was designed, cloned, and inserted into the pcDNA-SYVN1 vector to establish SYVN1 overexpression (SYVN1-OE) plasmids. When the cell density reached 50%, the pcDNA-SYVN1 plasmids were transfected through Lipofectamine 3000 (GenePharma, China). A Caspase-11 small interfering RNA (siRNA) was designed and transfected into MLE-12 cells. At 48 h after transfection, the medium was completely replaced. Then, 1-NP or subsequent processing was applied to the MLE-12 cells as indicated. At 24 h after 1-NP, the indicators of apoptosis and pyroptosis were detected.

### 2.7 Coimmunoprecipitation (Co-IP)

The interactions of Caspase-11 with SYVN1 and ubiquitin were determined using coimmunoprecipitation (Co-IP). Briefly, total protein was extracted from MLE-12 cells with cell lysis buffer after different treatments. The supernatant was obtained and some of it was used as input, while the remainder was used for the Co-IP. Equivalent amounts of cell lysate were incubated with magnetic beads A/G and different antibodies, including those against caspase-11, SYVN1 or ubiquitin at 4 °C for 36 h. The protein A/G beads were subsequently collected and boiled. The proteins were isolated and collected for detecting the interactions using Western blot.

### 2.8 Terminal deoxynucleotidyl transferase dUTP nick end labelling (TUNEL) assay

The number of apoptotic cells were evaluated in lung tissues and MLE-12 cells through an *in situ* cell death detection kit (Beyotime Biotechnology, China). In brief, lung sections or cell climbing films were fixed and permeabilized at 25 °C for 20 min. Subsequently, the sections or cell climbing films were incubated with fluorescein-conjugated TUNEL reaction reagent at 25 °C for 2.5 h in a light-free environment. Subsequently, the cell nuclei were stained. Fluorescence micrographs were obtained using a fluorescence microscope.

### 2.9 Isolation of RNA and real-time (RT)-polymerase chain reaction (PCR)

Total RNA was extracted from mouse lungs and MLE-12 cells via TRIzol reagent (Li M. D. et al., 2024). A total of 4 μg of RNA was transcribed into 30 μL of cDNA with a reverse transcription kit. RT‒PCR was performed via SYBR Green I master mix (Roche). The specific primers used were from Sagan Corporation (Shanghai, China) and are shown in Table 1. The comparative cycle threshold (2^−ΔΔCT^) method were used to estimate the levels of target genes.

**TABLE 1:** Oligonucleotide sequences.

Gene	Forward (5’-3’)	Reverse (5’-3’)	Species
*β-actin*	GCACCACACCTTCTACAAT	GTGAGGGAGAGCATAGCC	Mouse
*Il-18*	GACTCTTGCGTCAACTTCAAGG	CAGGCTGTCTTTTGTCAACGA	Mouse
*Il-1β*	ATCAACCAACAAGTGATATTCTCCAT	GGGTGTGCCGTCTTTCATTAC	Mouse
*Caspase-11*	ACAAACACCCTGACAAACCAC	CACTGCGTTCAGCATTGTTAAA	Mouse

### 2.10 Potential target genes identification and Kyoto Encyclopedia of Gene and Genome (KEGG) analysis

In order to the common target genes of 1-NP exposure and ALI, two public databases, GeneCards (https://www.genecards.org/) and CTD (https://ctdbase.org/) were used and analysed. The evident Differentially Expressed Genes (DEGs) were defined as the |log2 Fold Change| (log |FC| > 1.5) and adjusted P-value <0.05 with a threshold of P < 0.05. Adjusted P-values were calculated by Benjamini-Hochberg (BH) method to govern the False Discovery Rate (FDR), which avoids the random results from multiple testing. The target genes of 1-NP exposure and ALI were estimated by the Venny tool (https://bioinfogp.cnb.csic.es/tools/venny/index.html), and a Venn diagram was to explore the intersecting DEGs in the two data sets. The DEGs with logFC>0 was defined as increased genes, yet decreased genes were logFC<0. Kyoto Encyclopedia of Gene and Genome (KEGG) enrichment (https://metascape.org) was executed to distinguish the potential biological pathways of DEGs. Genes annotation, visualization, and integrated discovery function were analysed. The cutoff value was set as P < 0.05.

### 2.11 Statistical analysis

All statistical analyses were conducted using Statistical Package for the Social Sciences (SPSS) 19.0 software. The normality was analysed with Kolmogorov-Smirnov test. The normal-distributed data were expressed as means and non-normally distributed data were expressed as median. The data with normal distribution were compared with Student’s t-test or one-way analysis of variance (ANOVA) test. The parameters with nonnormal distribution were evaluated via Kruskal-Wallis H or Mann-Whitney U tests. The multiple comparisons were conducted with Newman-Keuls test. All the data are expressed as the means ± standard errors. *P* < 0.05 was considered as significant difference.

## 3 Results

### 3.1 Acute 1-NP induced ALI in mice

**FIGURE 1:**
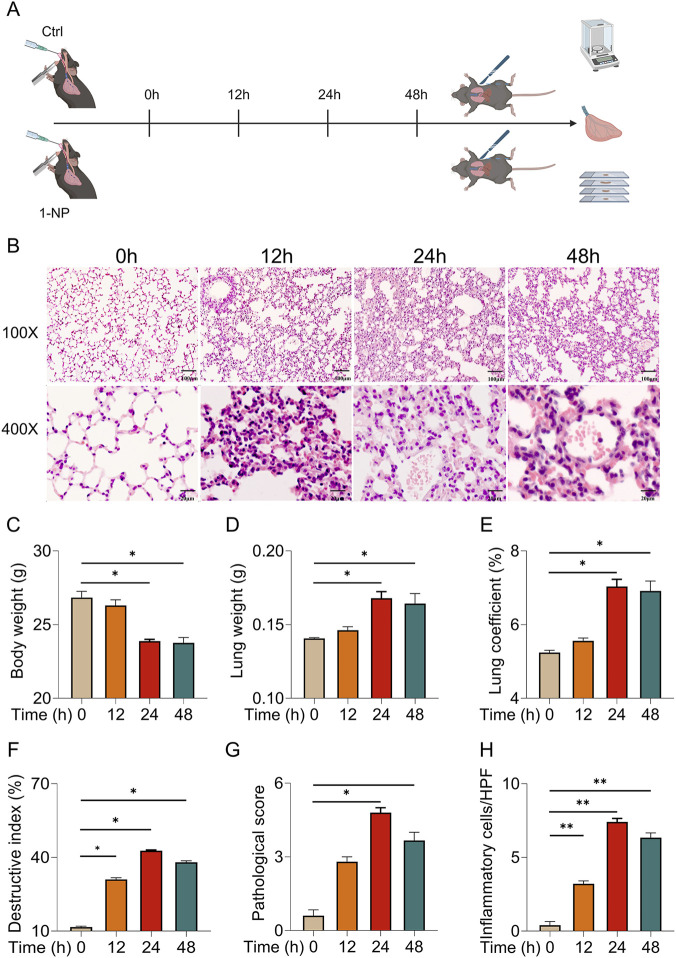
Acute 1-NP induced ALI in mice. **(A)** Flow chart of the animal experiment. **(B)** H&E staining. Original magnification: 100× and 400×. **(C)** Body weight. **(D)** Lung weight. **(E)** Lung coefficient. **(F)** Destructive index. **(G)** Pathological score. **(H)** Inflammatory cells. All data are displayed as means ± S.E.M.s of twenty samples. *P < 0.05, **P < 0.01.

Figure 1A represented the experimental scheme. H&E staining indicated that acute 1-NP provoked pathology injury and inflammatory cell infiltration (Figure 1B). In addition, 1-NP exposure decreased body weight from 24 h to 48 h. Inversely, the lung weight and lung coefficient were increased at 24 h and 48 h after 1-NP (Figures 1C–E). Moreover, the destructive index, pathological score, and number of inflammatory cells were elevated at 12, 24, and 48 h after 1-NP exposure (Figures 1F–H).

### 3.2 Acute 1-NP induced apoptosis in mouse lungs and MLE-12 cells

**FIGURE 2:**
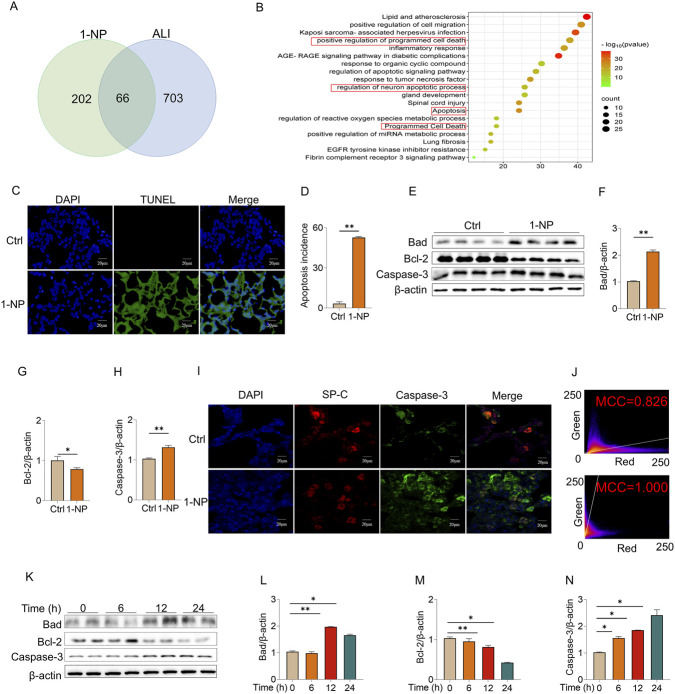
The effects of acute 1-NP on apoptosis in mouse lungs and MLE-12 cells. **(A)** The common genes associated with both ALI and 1-NP-evoked changes were explored by GeneCards and CTD databases. **(B)** Differential gene expression was analysed by KEGG. **(C,D)** Apoptotic cells were measured in mouse lungs by TUNEL. **(E–H)** Representative bands of apoptosis **(F)** and quantitative analyses of Bad **(F)**, Bcl-2 **(G)**, and Caspase-3 **(H)** expression. **(I,J)** The colocalization of SP-C with Caspase-3 was analysed by IF **(I)**, and quantitated by the Mander colocalization coefficient (MCC) **(J)**. **(K–N)** The influence of acute 1-NP on apoptosis was explored. **(K)** The expressions of apoptosis-related proteins were estimated with Western blot and quantitative analyses of Bad **(L)**, Bcl-2 **(M)**, and Caspase-3 **(N)**. All data are displayed as means ± S.E.M.s of six samples. The molecular experiments were repeated twice. *P < 0.05, **P < 0.01.

GeneCards and CTD predicted that there were 66 overlapping genes among the 1-NP-changed and ALI-related genes (Figure 2A). Further KEGG analyses suggested that the differential gene expression occurred mainly in the signalling pathways of programmed cell death and apoptosis (Figure 2B). The impacts of acute 1-NP on pyroptosis and apoptosis were subsequently determined in mouse lungs. TUNEL staining suggested that acute 1-NP increased the number of apoptotic cells in mouse lungs (Figures 2C,D). The protein expression of B cell lymphoma/leukemia 2 (Bcl-2) was decreased, and that of Bcl-xL/Bcl-2-associated death promoter (Bad) and Caspase-3 were increased after 1-NP exposure (Figures 2E–H). IF revealed that 1-NP repressed surfactant protein C (SP-C),a biomarker of pulmonary epithelial cells and highly hydrophobic protein found in pulmonary surfactant, and increased Caspase-3 (Figure 2I). 1-NP promoted the colocalization of SP-C with Caspase-3 in mouse lungs (Figure 2J). *In vitro* experiments suggested that acute 1-NP treatment upregulated Bad and Caspase-3, and inhibited Bcl-2 via a time-dependent manner (Figures 2K–N).

### 3.3 Acute 1-NP provoked pyroptosis in mouse lungs and MLE-12 cells

**FIGURE 3:**
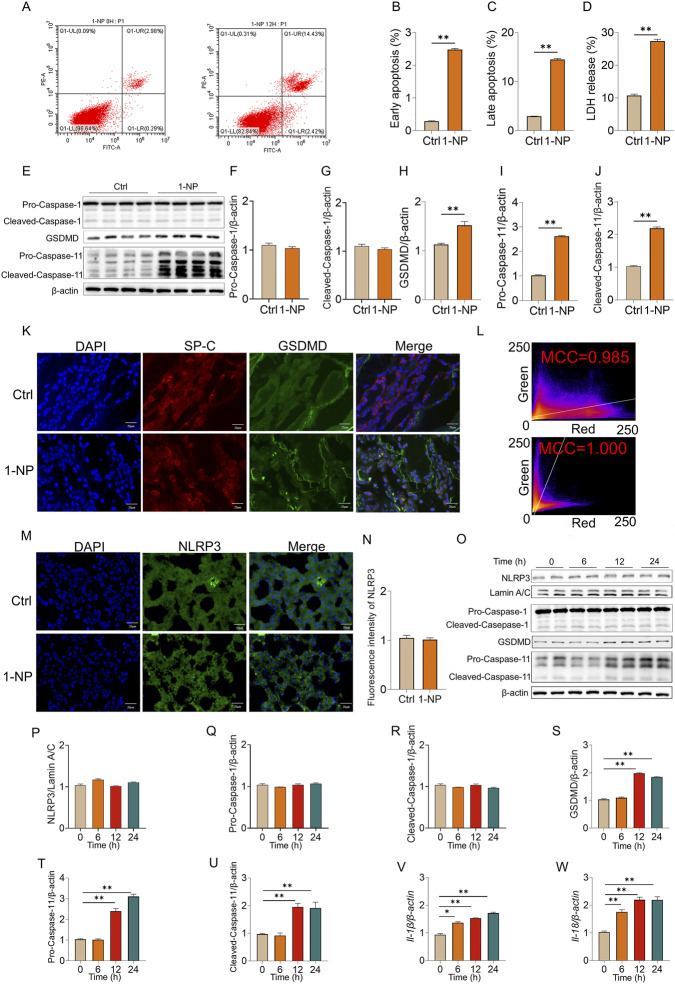
The effects of acute 1-NP on pyroptosis in mouse lungs and MLE-12 cells. **(A–C)** The number of apoptotic cells was detected in MLE-12 cells through flow cytometry **(A)**, and quantitative analyses of early apoptosis **(B)** and late apoptosis **(C)**. **(D)** Cytotoxicity was estimated by LDH release from MLE-12 cells. **(E–J)** The expressions of GSDMD, Pro-Caspase-11, Cleaved-Caspase-11, Pro-Caspase-1, and Cleaved-Caspase-1 were measured in mouse lungs by Western blot. **(K,L)** The co-localization between SP-C and GSDMD was determined in mouse lungs via IF. **(M,N)** NLRP3-positive nuclei were analysed in mouse lungs via IF and quantification. **(O–W)** The effect of acute 1-NP on pyroptosis was estimated in MLE-12 cells. **(O–U)** Representative bands of pyroptosis markers **(O)**, and the levels of NLRP3 **(P)**, Pro-Caspase-11 **(Q)**, Cleaved-Caspase-11 **(R)**, GSDMD **(S)**, Pro-Caspase-11 **(T)**, and Cleaved-Caspase-11 **(U)** were quantified. **(V,W)** The mRNAs of *Il-1β* and *Il-18* were determined in MLE-12 cells by RT‒PCR. All data are displayed as means ± S.E.M.s of six samples. The molecular experiments were repeated twice. *P < 0.05, **P < 0.01.

The early apoptosis and late apoptosis were increased (Figures 3A–C) and lactate dehydrogenase (LDH) release was elevated in 1-NP-treated MLE-12 cells via flow cytometry (Figure 3D), suggesting that acute 1-NP exposure provoked pyroptosis. Figures 3E–J showed that acute 1-NP elevated GSDMD, pro- and cleaved-Caspase-11 in mouse lungs, there was no obvious effect of 1-NP exposure on Pro- and Cleaved-Caspase-1. Moreover, 1-NP exposure aggravated the colocalization between GSDMD and SP-C in mouse lungs (Figures 3K,L). IF revealed that 1-NP did not affect the number of NOD-like receptor protein 3 (NLRP3)-positive nuclei in lung tissues (Figures 3M,N). Although 1-NP treatment did not change the expression of NLRP3 nuclear protein and Caspase-1 (Figures 3O–R), it significantly upregulated GSDMD and pro- and Cleaved-Caspase-11 in MLE-12 cells at 12 and 24 h (Figures 3O,S–U). The expressions of *interleukin (Il)-1β* and *Il-18* were increased from 6 h to 24 h after 1-NP (Figures 3V,W).

### 3.4 Pharmacological inhibition of Caspase-11 alleviated 1-NP-induced ALI

**FIGURE 4:**
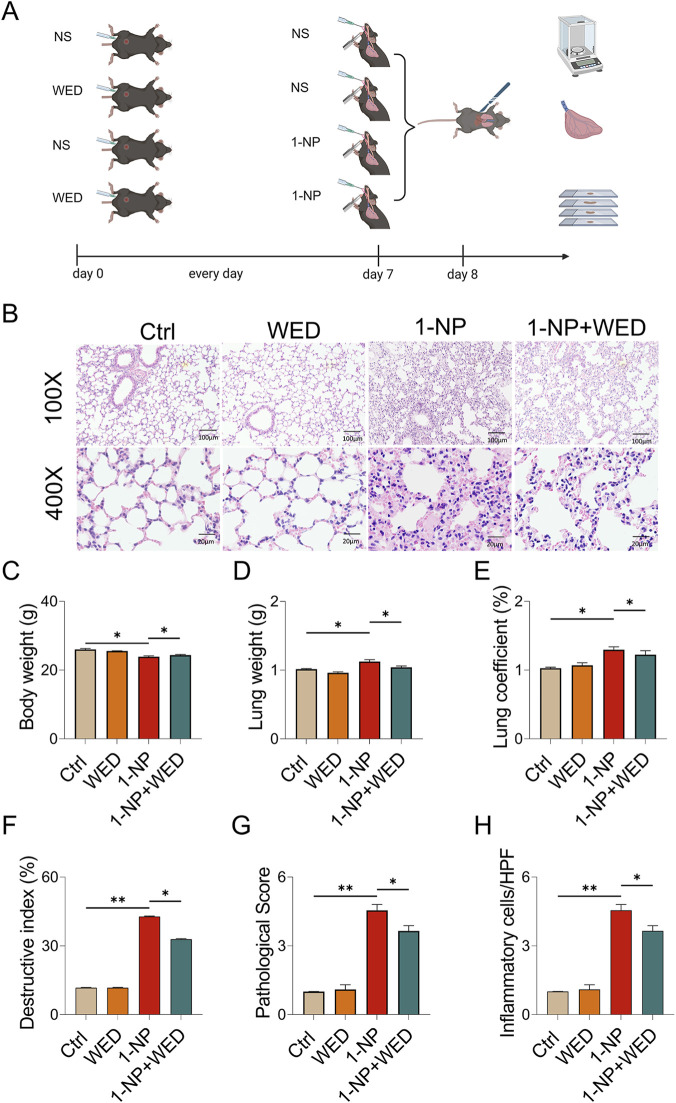
The effect of Caspase-11 upregulation on acute 1-NP-induced ALI in mice. **(A)** Flow chart of the animal experiment. **(B)** H&E staining. Original magnification: 100× and 400×. **(C)** Body weight. **(D)** Lung weight. **(E)** Lung coefficient. **(F)** Destructive index. **(G)** Pathological score. **(H)** Inflammatory cells. All data are displayed as means ± S.E.M.s of twenty samples. *P < 0.05, **P < 0.01.

To reveal the role of Caspase-11 activation on 1-NP-mediated ALI, the mice were pretreated with WED and then exposed to 1-NP (Figure 4A). The results suggested that WED pretreatment dramatically attenuated 1-NP-induced pulmonary pathology injury and inflammatory cell infiltration (Figure 4B). The 1-NP-induced decrease in body weight and increases in the lung weight and lung coefficient were inhibited by WED (Figures 4C–E). Moreover, WED supplementation inhibited the 1-NP-induced increases in the destructive index, pathological score, and number of inflammatory cells in mouse lungs (Figures 4F–H).

### 3.5 Pharmacological inhibition or genetic deletion of Caspase-11 abolished 1-NP-induced apoptosis

**FIGURE 5:**
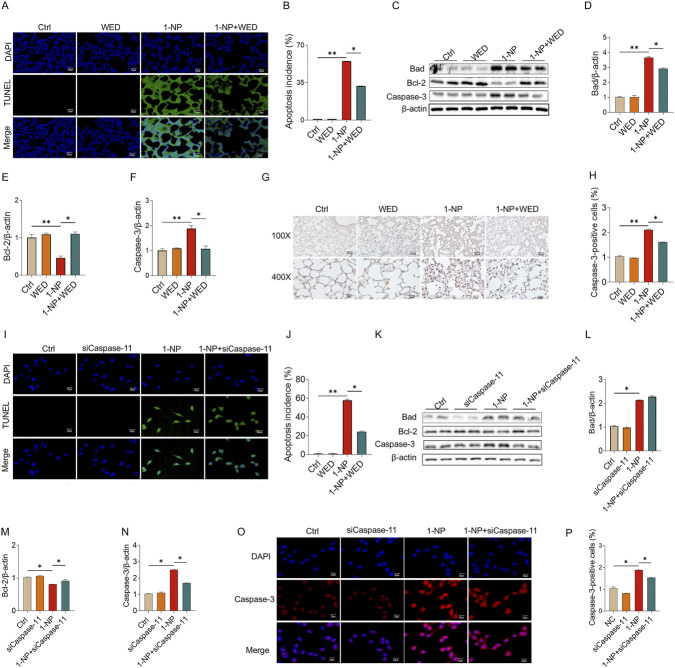
The influences of Caspase-11 elevation on 1-NP-evoked apoptosis in mouse lungs and MLE-12 cells. **(A–H)** The inhibitory effect of WED on 1-NP-incurred apoptosis was analysed in mouse lungs. **(A,B)** The number of apoptotic cells was evaluated via TUNEL. **(C–F)** The parameters of apoptosis were measured via Western blot, consisting of Bad, Bcl-2, and Caspase-3. **(G,H)** The number of Caspase-3-positive cells was determined by IHC. **(I–P)** The antagonistic influence of Caspase-11 siRNA on 1-NP-mediated apoptosis was explored in MEL-12 cells. **(I,J)** Apoptotic cells were detected using TUNEL. **(K–N)** Representative bands of apoptosis **(K)** and quantitative analyses of Bad **(L)**, Bcl-2 **(M)**, and Caspase-3 **(N)**. **(O,P)** The count of Caspase-3-positive cells was analysed. All data are displayed as means ± S.E.M.s of six samples. The molecular experiments were repeated twice. *P < 0.05, **P < 0.01.

The effect of Caspase-11 inhibition on 1-NP-triggered apoptosis was analysed in mouse lungs. TUNEL staining revealed that pretreatment with WED significantly abated the 1-NP-induced apoptosis (Figures 5A,B). The 1-NP-induced increases in Caspase-3 and Bad, as well as the decrease in Bcl-2, were dramatically inhibited by WED pretreatment (Figures 5C–F). Similarly, WED pretreatment also abolished the 1-NP-upregulated Caspase-3-positive cells in mouse lungs (Figures 5G,H). The influence of Caspase-11 siRNA on 1-NP-induced apoptosis was subsequently explored. Caspase-11 siRNA transfection notably decreased the incidence of apoptosis induced by 1-NP (Figures 5I,J). In addition, Caspase-11 siRNA transfection downregulated the 1-NP-mediated the changes in Bad, Bcl-2, and Caspase-3 in MLE-12 cells (Figures 5K–P).

### 3.6 Pharmacological inhibition or genetic deletion of Caspase-11 mitigated 1-NP-induced pyroptosis

**FIGURE 6:**
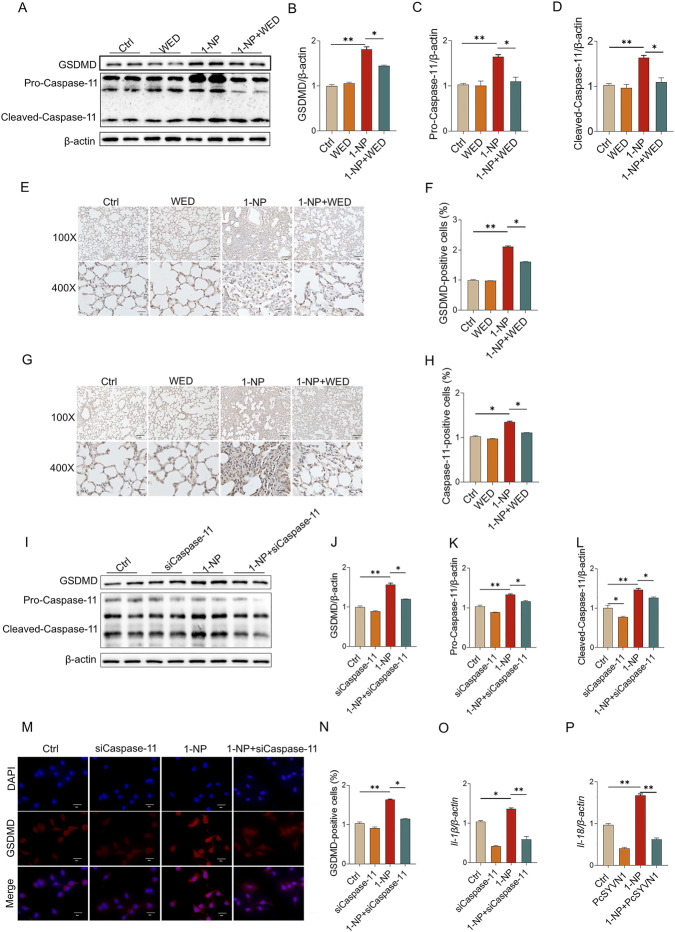
The effects of Caspase-11 increase on 1-NP-induced pyroptosis in mouse lungs and MLE-12 cells. **(A–H)** The antagonistic influence of WED on 1-NP-induced apoptosis was analysed in mouse lungs. **(A–D)** Pyroptosis markers were tested via Western blot, including GSDMD, Pro-Caspase-11, and Cleaved-Caspase-11. **(E,F)** GSDMD-positive cells were evaluated by IHC. **(G,H)** Caspase-11-positive cells were detected with IHC. **(I–P)** The repressive influence of Caspase-11 siRNA on 1-NP-induced pyroptosis was analysed in MLE-12 cells. **(I–L)** The indicators of pyroptosis were estimated using Western blot **(I)** and quantitative analyses of GSDMD **(J)**, Pro-Caspase-11 **(K)**, and Cleaved-Caspase-11 **(L)**. **(M,N)** The number of GSDMD-positive cells was evaluated using IF. **(O,P)** The mRNA levels of *Il-1β* and *Il-18* were estimated by RT‒PCR. All data are displayed as means ± S.E.M.s of six samples. The molecular experiments were repeated twice. *P < 0.05, **P < 0.01.

Pretreatment with WED effectively inhibited the 1-NP-mediated increases in GSDMD, pro- and cleaved Caspase-11 in mouse lungs (Figures 6A–D). In addition, the 1-NP-induced increases in GSDMA- and Caspase-11-positive cells in mouse lungs were prominently alleviated by WED pretreatment (Figures 6E–H). As expected, Caspase-11 siRNA transfection inhibited the 1-NP-induced upregulation of GSDMD, pro- and cleaved-Caspase-11 (Figures 6I–N), *Il-18* and *Il-1β* mRNAs in MLE-12 cells (Figures 6O,P).

### 3.7 Acute 1-NP exposure attenuated Caspase-11 proteasome degradation

**FIGURE 7:**
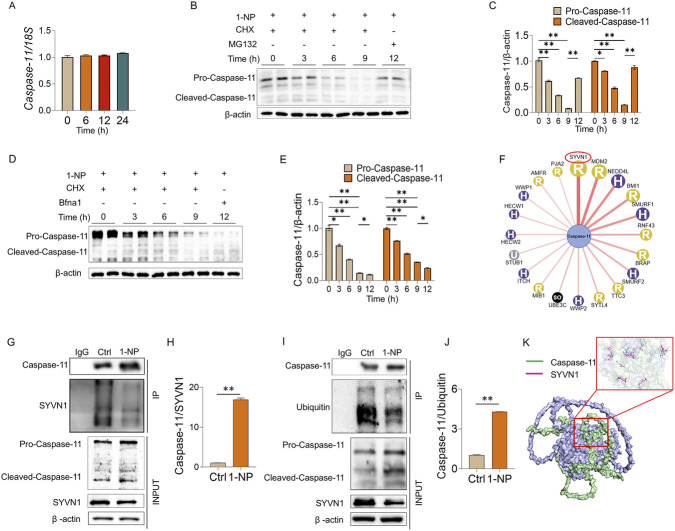
The impacts of acute 1-NP on Caspase-11 ubiquitination and protein degradation in pulmonary epithelial cells. **(A)** The level of *Caspase-11* mRNA was tested by RT‒PCR. At 24 h after 1-NP, MLE-12 cells were incubated with CHX or MG132 for 0 h, 3 h, 6 h, or 12 h. Caspase-11 protein stability was analysed. **(B,C)** The protein stability of Caspase-11 was estimated by Western blot. At 24 h after 1-NP, MLE-12 cells were incubated with CHX or Bnf A1 for different durations. **(D,E)** The protein stability of Caspase-11 was evaluated with Western blot. **(F)** The UbiBrowser database was used to predict the proteins that possibly interact with Caspase-11. **(G,H)** The interaction between Caspase-11 and SYVN1 was evaluated by Co-IP. **(I,J)** The interaction between Caspase-11 and ubiquitin was analysed via Co-IP. **(K)** The interaction between Caspase-11 and SYVN1 was simulated via molecular docking. All data are displayed as means ± S.E.M.s of six samples. The molecular experiments were repeated twice. *P < 0.05, **P < 0.01.

Acute 1-NP exposure did not affect *Caspase-11* mRNA in pulmonary epithelial cells (Figure 7A). CHX chase assays found that 1-NP suppressed Caspase-11 protein degradation in pulmonary epithelial cells (Figures 7B–E). Additionally, pretreatment with MG132, but not Baf A1, restored the 1-NP-induced suppression of Caspase-11 protein degradation by the ubiquitin–proteasome mechanism, but not by the autophagy–lysosome pathway (Figures 7B–E). The UbiBrowser database was used to predict proteins that may interact with Caspase-11. The top interacted protein was SYVN1, an E3 ubiquitin ligase (Figure 7F). Co-IP suggested that the 1-NP enhanced the interaction between Caspase-11 and SYVN1 (Figures 7G,H). Moreover, the interaction between Caspase-11 and ubiquitin was facilitated after 1-NP treatment (Figures 7I,J). Molecular docking also revealed a distinct interaction between Caspase-11 and SYVN1 (Figure 7K).

### 3.8 Acute 1-NP suppressed the ubiquitination degradation of Caspase-11 through inhibition of the E3 ubiquitin ligase SYVN1

**FIGURE 8:**
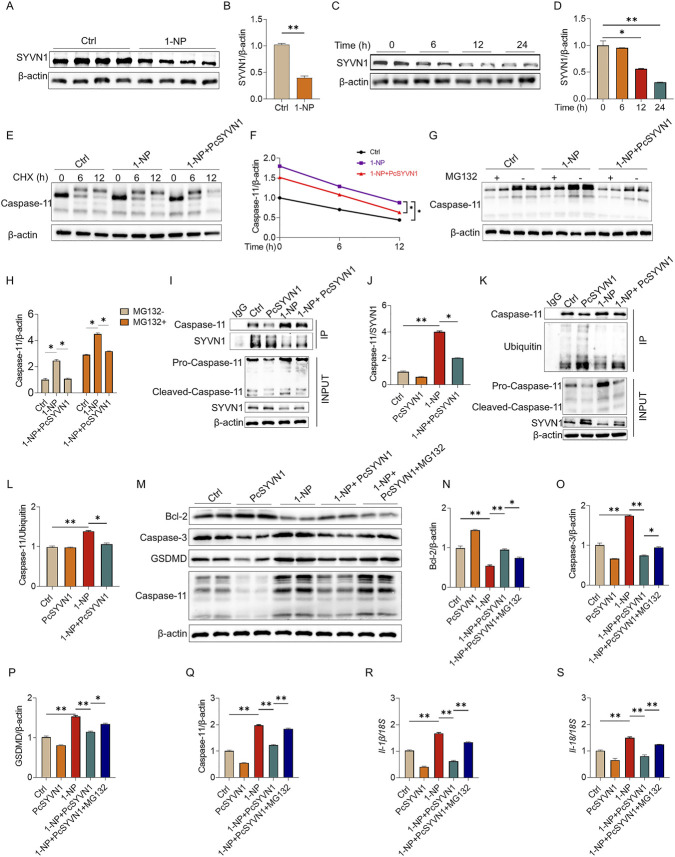
The role of SYVN1 on acute 1-NP-induced Caspase-11 proteasome degradation in pulmonary epithelial cells. **(A–D)** The effects of acute 1-NP on SYVN1 protein expression were evaluated in mouse lungs and MLE-12 cells via Western blot. **(E–L)** The effect of SYVN1 reduction on 1-NP-evoked Caspase-11 ubiquitination degradation was observed. SYVN1 overexpression plasmids were transfected, after which the cells were exposed to 1-NP (5 μM). **(E,F)** The stability of Caspase-11 protein was measured via Western blot. **(G,H)** The rate of Caspase-11 proteasome degradation was detected using Western blot. **(I,J)** The interaction between Caspase-11 and SYVN1 was analysed by Co-IP. **(K,L)** The interaction of Caspase-11 with ubiquitin was explored with Co-IP. **(M–S)** The effects of SYVN1 decrease on 1-NP-mediated apoptosis and pyroptosis were investigated in MLE-12 cells. **(M–Q)** The markers of apoptosis and pyroptosis were detected via Western blot. **(R,S)** The expressions of *Il-18* and *Il-1β* were evaluated through RT‒PCR. All data are displayed as means ± S.E.M.s of six samples. The molecular experiments were repeated twice. *P < 0.05, **P < 0.01.

Acute 1-NP exposure downregulated SYVN1 expression in mouse lungs and MLE-12 cells (Figures 8A–D). The antagonistic effect of SYVN1 overexpression on acute 1-NP-suppressed Caspase-11 proteasome degradation was evaluated. Transfection with SYVN1 overexpression plasmids decreased the stability of Caspase-11 (Figures 8E,F) by promoting Caspase-11 proteasome degradation (Figures 8G,H). In addition, SYVN1 overexpression blocked the 1-NP-induced the interaction between Caspase-11 and SYVN1, together with the interaction between Caspase-11 and ubiquitin (Figures 8I–L). As shown in Figures 8M–S, SYVN1 overexpression suppressed the 1-NP-induced decreases in Bcl-2 and increases in Caspase-3, GSDMD, Caspase-11, *Il-18* and *Il-1β* in MLE-12 cells. In addition, the influence of proteasome inhibition on the antagonistic effect of SYVN1 overexpression was explored. The results suggested that MG132 treatment attenuated SYVN1 overexpression-repressed 1-NP-mediated apoptosis and pyroptosis in MLE-12 cells (Figures 8M–S).

## 4 Discussion

The important findings mainly consisted of the following: First, acute 1-NP exposure induced ALI in mice; Second, acute 1-NP elicited pyroptosis and apoptosis in pulmonary epithelial cells through the activation of Caspase-11; Third, acute 1-NP exposure suppressed Caspase-11 ubiquitination and subsequent proteasome degradation; Fourth, acute 1-NP inhibited Caspase-11 proteasome degradation through the downregulation of the E3 ubiquitin ligase SYVN1; Fifth, pharmacological inhibition of Caspase-11 alleviated 1-NP-induced ALI. Our study provides evidence for environmental pollutant-induced ALI and new insights into drug development for treating ARDS in the future.

1-NP is widely present in the environment (Schauer et al., 2004; Gao et al., 2018). 1-NP can lead to pulmonary inflammation, pulmonary fibrosis, and COPD (Fu et al., 2021; Hu et al., 2020; Wang et al., 2025). However, before this study, the impacts of acute 1-NP on ALI and the underlying mechanisms were poorly understood. We found that acute 1-NP provoked alveolar destruction and pulmonary inflammation in mice. As expected, these pathological changes were consistent with other ALI models in mice induced by lipopolysaccharide (Xia et al., 2022), intestinal ischaemia/reperfusion (Ji et al., 2023), and sepsis (Zhang et al., 2022). These data demonstrated that acute 1-NP exposure successfully establishes an ALI model in mice. However, we must admit that the exposure dose of 1-NP (20 μg/mouse) in our investigation may be not inconsistent with the typical environmental exposure. The former study has uncovered that chronic exposure of 1-NP (145 μg) can evoke a COPD-like phenotype in mice within 4 months (Li et al., 2017). In addition, 1-NP exposure (20 μg/mouse/week) evokes pulmonary fibrosis in mice via intratracheal instillation after 15 weeks (Li S. R. et al., 2024). These data have suggested that chronic exposure to higher dose of 1-NP dramatically incurs chronic respiratory diseases. Due to ALI is an acute disease, acute 1-NP exposure is used to establish mice model. A latest research found that acute exposure to 1-NP (100 μg/mice) causes lung injury in mice (Kim et al., 2025). In order to explore the suitable dose of 1-NP acute exposure, the different dose of 1-NP (5; 10; 20; 50; 100 μg/mice) was treated in mice. The results suggested that the higher dose of 1-NP (50 μg; 100 μg/mice) obviously induced death in mice. On the contrary, the lower dose of 1-NP (5; 10 μg/mice) did n’t evoke evident pulmonary injury in mice. Of note, a single dose of 1-NP (20 μg/mice) via intratracheal instillation significantly incur ALI (Hu et al., 2020). Therefore, the dose of 1-NP (20 μg/mice) was selected and used in this study.

Apoptosis, as a type of programmed cell death, is implicated in various physiological processes of many diseases (Bertheloot et al., 2021). It’s known that apoptosis exerts a significant role in ALI (Zhu et al., 2023). Apoptosis is essential for eliminating abnormal cells via Bcl-2 and Caspase-3 signalling (Hentzen et al., 2020). The evident characteristics of apoptosis include cytochrome c release from mitochondria, an imbalance of antiapoptotic proteins, such as Bcl-xL and myeloid cell leukemia-1 (Mcl-1), and proapoptotic molecules, consisting of Bax and Bad, and increased activity of apoptotic executioners (Caspase-3) (Kumar et al., 2022). Animal experiments revealed that apoptotic cells were upregulated, the antiapoptotic protein Bcl-2 was decreased, and Bad and Caspase-3 were increased in mouse lungs after acute 1-NP inhalation. In addition, IF colocalization experiments indicated that 1-NP-evoked apoptosis in mouse lungs occurred mainly in pulmonary epithelial cells. Cellular experiments further confirmed that 1-NP coculture also led to MLE-12 apoptosis. These data hint that acute 1-NP results in ALI through inducing pulmonary epithelial cell apoptosis.

Moreover, increasing evidence has indicated that pyroptosis is the primary cause of the inflammatory reaction during ALI, which triggers injury to the plasma membrane and the secretion of extracellular inflammatory cytokines (Liu et al., 2022). From the present point of view, different pathways trigger pyroptosis, including the apoptotic Caspase-initiated pathway and the canonical and noncanonical inflammasome pathways (Rao et al., 2022). Inflammasomes are multimolecular complexes that contain NLRP3, apoptosis-associated speck-like protein containing a caspase recruitment domain (ASC), and the effector protease Caspase-1. Microbial components or infection quickly initiate inflammasome assembly and cleave Caspases into active forms (Rathinam and Fitzgerald, 2016; Broz and Dixit, 2016). GSDMD proteins are the ultimate executors of pore formation and lead to the release of inflammatory cytokines during pyroptosis. Caspases can mediate the innate immune response, provoke pyroptosis and then defend against pathogen invasion (Fernández and Lamkanfi, 2015). In our study, acute 1-NP exposure did not activate the NLRP3 inflammasome and Caspase-1, but increased the levels of Caspase-11 and GSDMD, and finally induced pyroptosis in pulmonary epithelial cells. The previous evidence has revealed that canonical pyroptosis is mediated by inflammasome assemble and Caspase-1 activation in an ASC-dependent manner (Xia et al., 2019). Moreover, in the non-canonical pathway, Caspase-4/5/11 are activated and cleaves GSDMD, and finally promotes pro-inflammatory cytokines production and incurs cellular proptosis (Liu et al., 2024). Many investigations have confirmed that the non-canonical pyroptosis pathway independent of NLRP3 is implicated in the progression of ALI (Tao et al., 2023; Chen et al., 2020). Moreover, Caspase-11 can directly cleave GSDMD and lead to pyroptosis independent of NLRP3-activated Caspase-1 (Kayagaki et al., 2015). Collectively, these data hinted that acute 1-NP may lead to ALI via noncanonical pyroptosis in pulmonary epithelial cells.

Overall, acute 1-NP exposure evoked ALI through the activation of pyroptosis and apoptosis. However, the exact mechanisms were elusive. Caspases can cleave substrates at aspartate residues and mediate apoptosis and inflammation (Nicholson DW, 1999). Different apoptotic stimuli initiate Caspases to cleave pro-Caspases (Caspase-3 and Caspase-7) and form bioactive Caspases. Caspase-3 is essential for promoting apoptotic cell death in the final stage (Eskandari and Eaves, 2022). In addition, as an inflammatory Caspase, Caspase-11 can be induced by apoptotic stimuli or stress. Caspase-11 upregulation can activate Caspase-3 and induce apoptosis (Miao et al., 2018; Kang et al., 2000). Moreover, there is evidence that noncanonical pyroptosis can be triggered by Caspase-11 (Kayagaki et al., 2011). Therefore, we speculated that Caspase-11-mediated apoptosis and pyroptosis are involved in the process of acute 1-NP-induced ALI. In the current study, acute 1-NP upregulated Caspase-11 in pulmonary epithelial cells. Additionally, gene knockdown of Caspase-11 clearly ameliorated 1-NP-induced pyroptosis and apoptosis in MLE-12 cells. Pharmacological inhibition of Caspase-11 also alleviated 1-NP-induced pyroptosis, apoptosis, and ALI in mouse lungs. These results strongly indicate that Caspase-11-mediated apoptosis and pyroptosis in pulmonary epithelial cells contribute, at least in part, to acute 1-NP-induced ALI.

Nevertheless, the mechanism by which Caspase-11 elevation is induced by acute 1-NP exposure was unclear. We found that acute 1-NP exposure inhibited Caspase-11 protein degradation. The ubiquitin–proteasome system and the autophagy–lysosome pathway are the foremost types of protein degradation (Ji et al., 2022). Further analysis indicated that acute 1-NP exposure repressed Caspase-11 protein degradation mainly by the ubiquitin–proteasome pathway, not the autophagy–lysosome pathway. Thus, we used the UbiBrowser database to explore the proteins that may interact with Caspase-11. We found that synoviolin (SYVN1) may interact with Caspase-11. SYVN1, an E3 ubiquitin ligase, can cause the degradation of endoplasmic reticulum-related proteins (Xu et al., 2019). The central roles of SYVN1 in ferroptosis and pyroptosis through regulating protein stability have been demonstrated in several investigations (Guo et al., 2023; Shi et al., 2022). We observed SYVN1 expression was downregulated, and the interaction between SYVN1 and Caspase-11 was increased after 1-NP. Moreover, SYVN1 overexpression dramatically attenuated the 1-NP induced suppression of proteasomal degradation of Caspase-11. Collectively, these data suggest that acute 1-NP may upregulate Caspase-11 expression and ALI via inhibiting SYVN1-mediated Caspase-11 proteasomal degradation in pulmonary epithelial cells. In this research, UbiBrowser database indicated there are many interacted proteins of Caspase-11, such as WWP1 (WW domain–containing E3 ubiquitin protein ligase 1), and WWP2, which are also E3 ubiquitin ligases and mediate a variety of proteins ubiquitination (Zhi and Chen, 2012; Zhang et al., 2020). However, only the effect of SYVN1, the top interacted protein of Caspase-11, on 1-NP-induced Caspase-11 upregulation was determined. The potential influences of other E3 ubiquitin ligases and deubiquitinating enzymes on 1-NP-repressed Caspase-11 ubiquitination degradation can’t be eliminated in the current study. In addition, although SYVN1 overexpression alleviated 1-NP-mediated apoptosis and pyroptosis in pulmonary epithelial cells, the impact of SYVN1 on 1-NP-incurred ALI was unclear in mice. So, more experiments should be conducted to determine the function of SYVN1 on 1-NP-mediated ALI.

This study suggested that acute 1-NP induced ALI through Caspase-11-evoked apoptosis and pyroptosis in pulmonary epithelial cells. We must acknowledge that there were some limitations in the current research. First, several forms of regulated cell death have been indicated to be implicated in the pathogenetic progress of ALI, such as pyroptosis, apoptosis, autophagy (Kong et al., 2021), ferroptosis (Wu et al., 2024), necroptosis (Sha et al., 2024), cuproptosis (Hou et al., 2024). The other studies have found that 1-NP exposure induces mitophagy (Li J. et al., 2024) and ferroptosis (Yu et al., 2025). Our group also revealed that 1-NP exposure activates nuclear factor-kappa B (NF-κB) singling in pulmonary epithelial cells (Hu et al., 2020). Due to the limitation of the research design, not all forms of cell death and inflammatory singling pathways could be clearly demonstrated in one report. The goal of this investigation was to explore the roles of proptosis and apoptosis on 1-NP-incurred ALI. Therefore, the influences of the other different forms of cell death, such as ferroptosis and cuproptosis, and inflammatory singling pathways could n’t be ruled out in the current research. More forms of regulated cell death would be evaluated on the process of 1-NP-triggered ALI in the future. Second, whether Caspase-11 activated apoptotic pathways during the process of 1-NP-mediated ALI depended on Caspase-3 is not completely clear. Pharmacological inhibition or genetic deletion of Caspase-3 should be performed to confirm the role of Caspase-3 in Caspase-11-mediated cellular apoptosis during 1-NP-triggered ALI in the future. Third, only one dose of 1-NP was selected for the acute exposure in the animal experiment. The selected dose of 1-NP is slightly greater than that expected to be experienced by the human body. The effects of low-dose and chronic exposure to 1-NP on pyroptosis and apoptosis are unknown. Other doses and different durations of exposure to 1-NP will be used in the subsequent experiments. Fourth, the influence of SYVN1 overexpression on 1-NP-mediated Caspase-11 proteasome degradation was explore in MLE-12 cells. But, due to the limitations of experimental conditions and there isn’t specific agonist of SYVN1, the impacts of SYVN1 overexpression on 1-NP-triggered ALI can’t be estimated in mice within a short time.

## 5 Conclusion

Overall, our findings showed that acute 1-NP induces ALI via Caspase-11-mediated apoptosis and pyroptosis in pulmonary epithelial cells. Further mechanistic analysis revealed that a decrease in SYVN1 is involved in acute 1-NP-induced suppression of Caspase-11 proteasomal degradation in pulmonary epithelial cells. Our research confirmed that acute exposure to environmental pollutants can lead to ALI. In addition, our data provide new insight for ALI therapy and reveal a novel mechanism by which SYVN1 regulates Caspase-11-mediated apoptosis and pyroptosis during ALI induction.

## Data Availability

The raw data supporting the conclusions of this article will be made available by the authors, without undue reservation.

## Glossary

ALI: Acute lung injury
ARDS: Acute respiratory dysfunction syndrome
ATCC: American Type Culture Collection
ASC: Apoptosis-associated speck-like protein containing a caspase recruitment domain
Bad: Bcl-xL/Bcl-2-associated death promoter
Baf A1: Bafilomycin A1
Bcl-2: B cell lymphoma/leukemia 2
Caspase: Cysteine aspartate-specific protease
Co-IP: Coimmunoprecipitation
CHX: Cycloheximide
Ctrl: Control
DMEM: Dulbecco’s Modified Eagle Medium
FBS: Fetal bovine serum
GSDMD: Gasdermin D
IF: Immunofluorescence
IHC: Immunohistochemistry
Il-1β: Interleukin-1β
Il-18: Interleukin-18
LDH: Lactate dehydrogenase
NF-κB: Nuclear factor-kappa B
NLRP3: NOD-like receptor protein 3
MLE-12: Mouse lung epithelial
1-NP: 1-Nitropyrene
PVDF: Polyvinylidene fluoride
RT-PCR: Real-time -polymerase chain reaction
siRNA: Small interfering RNA
SYVN1: Synoviolin
SPSS: Statistical Package for the Social Sciences
SP-C: Surfactant proteins C
TUNEL: Terminal deoxynucleotidyl transferase dUTP nick end labelling
WED: Wedelolactone.
