# Supplementary material for: 1-Nitropyrene induces acute lung injury via SYVN1/Caspase-11-mediated apoptosis and pyroptosis in pulmonary epithelial cells
Source: Front Pharmacol. 2026 Feb 9;17:1723593. doi: 10.3389/fphar.2026.1723593 (PMC12926500; doi:10.3389/fphar.2026.1723593)
Supplement: Supplementary file 1 [file Table1.docx]

**Supplemental Table 1. The primary antibodies, chemicals and critical reagent kits.**

| Antibodies | Source | Identifier |
| --- | --- | --- |
| Anti-β-actin | Cell Signaling Technology | Cat#8H10D10 |
| Anti-Lamin A/C | Santa Cruz | Cat#sc-7292 |
| Anti-Bad | Abcam | Cat#ab32445 |
| Anti-Bcl-2 | Abcam | Cat#ab182858 |
| Anti-Caspase-3 | Cell Signaling Technology | Cat#9662 |
| Anti-Caspase-1 | Invitrogen | Cat#MA5-16215 |
| Anti-Caspase-11 | Abcam | Cat#ab180673 |
| Anti-NLRP3 | Abcam | Cat#ab263899 |
| Anti-GSDMD | Abcam | Cat#ab219800 |
| Anti-SYVN1 | Abcam | Cat#ab170901 |
| Anti-Ubiquitin | Abcam | Cat#ab134953 |
| Anti-SP-C | Proteintech | Cat#10774-1-AP |
